# Safety of Lecanemab After 6‐Month Treatment in the DIAN‐TU‐001 Trial

**DOI:** 10.1002/alz70859_105304

**Published:** 2025-12-25

**Authors:** Jin Zhou, Erica Andreozzi, Jorge J. Llibre‐Guerra, David B. Clifford, David Li, Guoqiao Wang, Lon S. S Schneider, Eric McDade, Larisa Reyderman, Randall J. Bateman

**Affiliations:** ^1^ Eisai Inc., Nutley, NJ USA; ^2^ Washington University School of Medicine, St. Louis, MO USA; ^3^ Washington University School of Medicine, St Louis, MO USA; ^4^ Keck School of Medicine of USC, Los Angeles, CA USA; ^5^ Washington University School of Medicine in St. Louis, St. Louis, MO USA

## Abstract

**Background:**

The Dominantly Inherited Alzheimer Network Trials Unit (DIAN‐TU) trial is an adaptive platform trial evaluating various therapies intended to slow or prevent the Alzheimer disease (AD) progression in Dominantly Inherited Alzheimer’s disease (DIAD). In the Tau NexGen DIAN‐TU‐001 arm, the potential benefits of the anti‐tau therapy etalanetug (E2814), a novel monoclonal antibody that binds to the MTBR, are being investigated with the anti‐amyloid treatment lecanemab given as background therapy. While lecanemab has been shown to be well tolerated in multiple clinical trials, known risks include amyloid‐related imaging abnormalities (ARIA) and infusion reactions. Herein, we provide an overview of 6‐month safety results for DIAD participants in the symptomatic cohort (CDR 0.5‐1, n=97) receiving lecanemab only in the Tau NexGen DIAN‐TU‐001 Trial.

**Methods:**

DIAN‐TU‐001 is a phase II/III multicenter randomized, double‐blind, placebo‐controlled platform trial of potential disease modifying therapies utilizing biomarker, cognitive, and clinical endpoints in DIAD. Safety evaluations included monitoring of vital signs, physical examinations, adverse events, clinical laboratory parameters, and 12‐lead electrocardiograms. ARIA occurrence was monitored throughout the study by MRI, read both locally and centrally.

**Preliminary Results:**

Lecanemab was generally well‐tolerated during the 6‐month lecanemab‐only treatment period. Overall, 80.4% of the 97 participants enrolled and treated experienced an adverse event. The most common AEs were ARIA‐H (26.8%), headache (25.8%), ARIA‐E (20.6%), and infusion‐related reactions (11.3%). The incidence of ARIA‐E was 20.6% and the incidence of ARIA‐H was 26.8%. The rate of symptomatic ARIA‐E was 4.1% and symptomatic ARIA‐H was 1.0%. ARIA‐E most commonly occurred within the first 3 months of treatment (80.0%) and resolved within 4 months (75%). There were no cases of symptomatic isolated ARIA‐H, no intracerebral hemorrhage (>10 mm), and 2 (2.06%) individuals who experienced ARIA leading to lecanemab withdrawal. The majority AEs were mild or moderate, with a total of 6 (6.19%) participants experiencing a serious AE.

**Conclusions:**

Lecanemab was generally well‐tolerated. The most common adverse events of lecanemab in the DIAD population were ARIA‐H, headache, ARIA‐E, and infusion‐related reactions. DIAD population may have a higher rate of cerebral amyloid angiopathy with ARIA rates between heterozygotes and homozygotes in sporadic AD.

